# Familial Hypercholesterolemia Genetic Variations and Long-Term Cardiovascular Outcomes in Patients with Hypercholesterolemia Who Underwent Coronary Angiography

**DOI:** 10.3390/genes12091413

**Published:** 2021-09-14

**Authors:** Wen-Jane Lee, Han-Ni Chuang, Yi-Ming Chen, Kae-Woei Liang, Hsin Tung, Jun-Peng Chen, I-Te Lee, Jun-Sing Wang, Ching-Heng Lin, Hsueh-Ju Lin, Wayne Huey-Herng Sheu, Wen-Lieng Lee, Tzu-Hung Hsiao

**Affiliations:** 1 Department of Medical Research, Taichung Veterans General Hospital, Taichung 40705, Taiwan; wjlee@vghtc.gov.tw (W.-J.L.); hannichuang@alum.ccu.edu.tw (H.-N.C.); blacklark@gmail.com (Y.-M.C.); epid@ms39.hinet.net (C.-H.L.); cheryllin520@gmail.com (H.-J.L.); 2 Department of Social Work, Tunghai University, Taichung 40705, Taiwan; 3 Division of Allergy, Immunology and Rheumatology, Department of Internal Medicine, Taichung Veterans General Hospital, Taichung 40705, Taiwan; 4 Cardiovascular Center, Taichung Veterans General Hospital, Taichung 40705, Taiwan; ekwliang@gmail.com; 5 Department of Medicine, School of Medicine, National Yang-Ming University, Taipei 11221, Taiwan; itlee@vghtc.gov.tw (I.-T.L.); jswang@vghtc.gov.tw (J.-S.W.); whhsheu@vghtpe.gov.tw (W.H.-H.S.); 6 Center of Faculty Development, Taichung Veterans General Hospital, Taichung 40705, Taiwan; s19001009@gmail.com; 7 Biostatistics Task Force of Taichung Veterans General Hospital, Taichung 40705, Taiwan; pippan7676@vghtc.gov.tw; 8 Division of Endocrinology and Metabolism, Department of Medicine, Taichung Veterans General Hospital, Taichung 40705, Taiwan; 9 National Defense Medical Center, School of Medicine, Taipei 11490, Taiwan; 10 Department of Medicine, School of Medicine, Chung Shan Medical University, Taichung 40201, Taiwan; 11 Rong Hsing Research Center for Translational Medicine, Institute of Biomedical Science, College of Life Science, National Chung Hsing University, Taichung 40227, Taiwan; 12 Ph.D. Program in Translational Medicine, National Chung Hsing University, Taichung 40705, Taiwan; 13 Department of Industrial Engineering and Enterprise Information, Tunghai University, Taichung 40705, Taiwan; 14 Department of Public Health, College of Medicine, Fu Jen Catholic University, New Taipei City 242062, Taiwan; 15 Institute of Public Health and Community Medicine Research Center, National Yang-Ming University, Taipei 11221, Taiwan; 16 Institute of Biomedical Sciences, National Chung Hsing University, Taichung 40227, Taiwan; 17 Division of Endocrinology and Metabolism, Department of Medicine, Taipei Veterans General Hospital, Taipei 11217, Taiwan; 18 Institute of Genomics and Bioinformatics, National Chung Hsing University, Taichung 40227, Taiwan

**Keywords:** coronary artery disease, mortality, familial hypercholesterolemia, gene

## Abstract

Background: Familial hypercholesterolemia (FH) has been associated with early coronary artery disease (CAD) and increased risk of atherosclerotic cardiovascular disease. However, the prevalence of FH and its long-term outcomes in a CAD-high-risk cohort, defined as patients with hypercholesteremia who underwent coronary angiography, remains unknown. Besides, studies regarding the impact of genetic variations in FH on long-term cardiovascular (CV) outcomes are scarce. Methods and Results: In total, 285 patients hospitalized for coronary angiography with blood low-density lipoprotein cholesterol (LDL-C) levels ≥ 160 mg/dL were sequenced to detect FH genetic variations in LDL receptors apolipoprotein B and proprotein convertase subtilisin/kexin type 9. Risk factors associated with long-term CV outcomes were evaluated. The prevalence of FH was high (14.4%). CAD and early CAD were significantly more prevalent among FH variation carriers than non-carriers, despite comparable blood LDL-C levels. Moreover, the FH variation carriers also underwent more revascularization after a mean follow-up of 6.1 years. Multivariate logistic regression demonstrated that FH genetic variation was associated with increased incidence of cardiovascular disease and mortality (odds ratio = 3.17, *p* = 0.047). Two common FH variants, *LDLR* c.986G>A and *LDLR* c.268G>A, showed the most significant impacts on high blood LDL-C levels and early-onset CAD. Conclusions: Our results indicate that FH genetic variants may exhibit differential effects on early-onset CAD and revascularization risks in patients undergoing coronary angiography. FH genetic information might help identify high-risk patients with typical CAD symptoms for appropriate intervention.

## 1. Introduction

Familial hypercholesterolemia (FH) is an autosomal dominant disease mainly caused by pathogenic variants of the genes involved in cholesterol metabolism, resulting in impaired clearance of circulating low-density lipoprotein cholesterol (LDL-C). The prevalence of heterozygous FH in the general Caucasian population has been reported to be approximately 1 in 200–500 persons [1,2] and approximately 1 in 500 persons in Han Chinese, including Taiwanese [1,3]. Owing to the cumulative lifelong exposure to high blood LDL-C levels, individuals with FH have been clinically associated with a high risk of early-onset atherosclerotic cardiovascular disease (ASCVD) [4,5] and ischemic stroke [6,7].

Accumulating genetic data have shown that most FH cases result from a heterozygous pathogenic variant in three different genes which encode key proteins involved in the LDL receptor endocytic and recycling pathways, including the LDL receptor (*LDLR*), apolipoprotein B (*APOB*), and proprotein convertase subtilisin kexin 9 (*PCSK9*) [1,8]. Moreover, substantial studies have examined the high prevalence of FH in patients with coronary artery disease (CAD; 9.7–9.4%) compared with the general population [9,10], based on the clinical criteria. In young myocardial infarction (MI) cases, the prevalence of genetically confirmed FH was 1.3%, which increased sixfold as compared with that in the general population of the United Kingdom [11]. FH prevalence also increased fivefold in the CAD population in Japan [12]. In early-onset CAD, the FH prevalence further increased 15-fold [13].

Hypercholesterolemia is a major risk factor for CAD. The meta-analysis of statin trials showed that CAD-associated morbidity and mortality could be efficiently decreased by 16% with intensive cholesterol-lowering therapy compared with the standard statin therapy [14]. Furthermore, every 1 mmol/l increase in LDL level was reported to be related to a 1.45 increase in the risk ratio of MI in patients with genetic alterations [15]. Moreover, FH dramatically increased the early CAD risk by least 13- to 22-fold without treatment [16]. However, to our knowledge, whether FH also influences the clinical outcomes after revascularization in patients with signified CAD, especially those with hypercholesterolemia, remains unclear. In this study, we examined the prevalence of FH and the effects of genetic variants on the lipid profile and clinical presentations of patients with hypercholesterolemia who underwent coronary angiography. This study is the first to follow up a CAD-high-risk cohort for up to 10 years in a Han Chinese population.

## 2. Materials and Methods

### 2.1. Study Population

From January 2010 to January 2020, 6920 patients suspected of having acute coronary syndrome were admitted to Taichung Veterans General Hospital to undergo coronary angiography. Approximately 8% of the patients had high blood cholesterol levels, defined as LDL-C levels ≥ 160 mg/dL. Among the patients, 285 subjects were randomly selected and underwent genetic analysis for FH. This study was approved by the Human Research Review Committee of Taichung Veterans General Hospital, Taichung, Taiwan. Informed written consent was also obtained from all of the subjects enrolled in this study. All of the study protocols were in accordance with the Declaration of Helsinki.

### 2.2. Data Collection and Follow-up

The baseline characteristics (age, sex, body height, body weight, waist and hip circumferences, and blood pressure), clinical and biochemical data (the levels of lipids, glucose, hemoglobin A1c [HbA1c], creatinine, etc.), medical history (diabetes mellitus [DM], hypertension, CAD, etc.), angiographic images, catheterization reports, medication use (statin [HMG-CoA reductase inhibitors], etc.), and medical chart records of all of the 285 subjects were collected and reviewed. DM was defined as a fasting blood glucose level ≥ 126 mg/dL on two occasions or current intake of anti-diabetic medication. Hypertension was defined as a systolic blood pressure (sBP) ≥ 140 mmHg or a diastolic blood pressure (dBP) ≥ 90 mmHg or current intake of antihypertensive medication. CAD was defined as having any one of the following conditions: acute or old MI, ≥50% stenosis on coronary angiography, percutaneous coronary intervention (PCI), or coronary artery bypass grafting (CABG). Early CAD was defined as an age of CAD onset of ≤45 years for men and ≤55 years for women [17].

Moreover, a 10-year follow-up was conducted in all of the 285 enrolled patients using the hospital-based electronic health record system. The impact of FH genetic variation on the long-term cardiovascular (CV) outcomes was evaluated based on the presence of major adverse cardiac events (MACEs), defined as a composite of all-cause death, nonfatal myocardial infarction, or nonfatal stroke. Revascularization, defined as having PCI or CABG, on follow-up was also evaluated.

### 2.3. Targeted Sequencing

A 2-mL peripheral blood sample was collected from each subject for genomic DNA extraction. Genomic DNA was extracted from leukocytes using a QIAamp DNA Blood Mini Kit (Qiagen, Hilden, Germany) for subsequent next-generation sequencing analysis. Targeted sequencing was used to sequence the target area of interest linked to FH, including only the whole exons of the *LDLR*, *APOB*, and *PCSK9* genes. Probes/primers specific these genes were designed. The targeted panel used was the FH gene test assay used for clinical genetic trials in the Precision Medicine Laboratory of Taichung Veterans General Hospital. Polymerase chain reaction was performed to amplify and then sequence the candidate DNA fragments. Library construction was performed using a Qiagen target panel kit (Qiagen, CDHS-15658z-227, Hilden, Germany) and was then quantified. The prepared library was then loaded onto the Illumina Sequencing System (iSeq 100/MiniSeq, San Diego, CA, USA). The final library concentration was 40 pM (iSeq 100) or 1 pM (MiniSeq). The sequence experiment was performed in accordance with the QIASeq Targeted DNA Panel Handbook. The FastQ files from the targeted DNA libraries were stored in CLC Genomics Workbench 12 (QIAGEN, Demark), and variants calling further performed by QIAGEN Panel analyses. The assessment of variants’ pathogenicity was performed with the Illumina Basespace Variant Interpreter. The pathogenicity (pathogenic or likely pathogenic variants) was next confirmed with the ClinVar database. The ClinVar database is a public archive providing information on human genomic variants that have been interpreted for their relationships to diseases with supporting evidence of clinical or functional significance.

### 2.4. Statistical Analyses

Continuous variables were expressed as their mean ± standard deviation (SD), while categorical variables were expressed as numbers (percentages). An independent *t*-test was used to analyze the continuous variables, and the chi-square test or Fisher exact test was used for the categorical variables. Furthermore, univariate and multivariate logistic regression analyses (enter method) were performed to evaluate the effects of FH genetic variation on the incidences of cardiovascular disease (CVD) and mortality. Statistical analyses were performed using the Statistical Package for Social Sciences (IBM SPSS version 22.0; International Business Machines Corp, New York, NY, USA). A two-tailed *p*-value < 0.05 was considered statistically significant.

## 3. Results

### 3.1. High Prevalence of FH in Patients with High Blood LDL-C Levels Who Underwent Coronary Angiography

Forty-one of the 285 study patients were identified as FH based on detected pathogenic/likely pathogenic variants on their *LDLR*, *APOB*, or *PCSK9* gene, which equated to a prevalence as high as 14.4%. The clinical and biochemical characteristics of the study cohort, stratified according to the genetic diagnosis of FH, were summarized in Table 1 (left panel). We found that the prevalence rates of CAD and early-onset CAD were significantly higher among the FH variant carriers (87.8% vs. 71.7%, *p* = 0.048 and 26.8% vs. 9.8%, *p* = 0.005, respectively). Moreover, 90.9% (10/11) of the early CAD cases were male. In addition, we also analyzed this cohort with LDL-C ≥ 190 mg/dL (The Dutch Lipid Clinical Network [DLCN] criteria for diagnosing possible FH), the result pattern was similar (Supplementary Table S1).

### 3.2. FH Pathogenica Vatiants on LDLR, APOB, and PCSK9

A total of 16 FH genetic variants were detected in 41 subjects. Forty subjects carried a heterozygous variant, and only one subject was a double heterozygous variant carrier (*LDLR* c.1867 A>G and *APOB* c.10579 C>T). Among the variants, 13 were missense single nucleotide variants (SNVs), two were splicing region SNVs, and one was a frameshift deletion/insertion (Indel). Details are shown in Table 2.

As expected, most FH genetic variants (85.7%, 36/42) were identified on *LDLR*, including 10 missense SNVs, two splicing region SNVs, and one frameshift Indel. Moreover, the top four variants, namely *LDLR* c.1747C>T, *LDLR* c.986G>A, *LDLR* c.268G>A, and *LDLR* c.1867A>G, accounted for more than half (57.1%, 24/42) of the *LDLR* variants. By contrast, only two missense SNVs were detected on the *APOB* gene, which accounted for 11.9% (5/42) of the variants, with one common variant, *APOB* c.10579C>T (9.5%, 4/42), and one rare variant, *APOB* c.10700C>T (2.4%, 1/41). In addition, one rare *PCSK9* c.658G>A variant was identified in one of our study subjects.

### 3.3. Association of FH Pathogenic Variants with the Incidence of CVD or Mortality

To evaluate the impact of FH pathogenic variants on the incidence of CVD or mortality, a 10-year follow-up was conducted among patients in the cohort. After a mean follow-up of 6.1 ± 3.1 years, 55 study subjects (19.3%) experienced MACEs, which resulted in all-cause death in 41 patients (14.4%), nonfatal MI in 15 patients (5.3%), and nonfatal stroke in 5 patients (1.8%). Supplementary Table S2 summarizes the MACEs in the cohort, stratified according to the genetic diagnosis of FH. No significant difference in the incidence of MACEs was found between the FH pathogenic variant carriers (n = 41) and non-carriers (n = 244). In addition, 75 patients (26.3%) were found to have revascularization (PCI or CABG) on follow-up (Table 3, left panel). As expected, the FH variant carriers had significantly more revascularization on follow-up than the non-carriers (51.2% vs. 22.1%, *p* < 0.001), especially those who received CABG (19.5% vs. 2.0%, *p* < 0.001).

The univariate logistic regression analysis revealed that eight indicators, namely male sex, smoking habit, low high-density lipoprotein cholesterol (HDL-C) level, high LDL-C level, low estimated glomerular filtration rate (eGFR), DM, hypertension, and FH genetic variation, were associated with the incidence of CVD or mortality (Table 4). Even after adjustment for sex, smoking, blood pressure, LDL-C level, eGFR, and DM, the multivariate logistic regression analysis revealed that the FH genetic variation was associated with the highest incidence of CVD or mortality (odds ratio [OR] = 3.17, *p* = 0.047), which implied the strong impact of the FH genetic variation.

### 3.4. Association of the LDLR c.986G>A and LDLR c.268G>A Variants with High Blood LDL-C Levels and Early-Onset CAD

We further focused on the five FH genetic variants with high allele frequencies in carriers to examine the impact on blood LDL-C level and its associated CV risk. We found that carriers with either the *LDLR* c.986G>A (n = 5) or *LDLR* c.268G>A variant (n = 4) had higher blood LDL-C levels (214 ± 25 mg/dL vs. 212 ± 29 mg/dL) than the other three variants (Supplementary Table S3). In the combined analysis, Table 1 (right panel) demonstrated that the carriers of *LDLR* c.986G>A/*LDLR* c.268G>A (n = 9) had significantly higher blood LDL-C levels than the FH variant non-carriers (n = 244; 213 ± 25 mg/dL vs. 190 ± 30 mg/dL, *p* = 0.026). Moreover, the mean age of the men with either of the two variants was significantly younger than that of others (47.3 ± 11.9 years vs. 58.4 ± 12.1 years, *p* = 0.028), especially the carriers of *LDLR* c.268G>A (39.0 ± 1.7 years, Supplementary Table S3). In addition, more than half of the carriers of the two variants (5/9), all of whom were male, had early-onset CAD as compared with the others (55.6% vs. 9.8%, *p* = 0.001).

We also investigated the onset age of CAD between the two groups. As shown in Supplementary Figure S1, the age of CAD onset in the carriers of *LDLR* c.986G>A/*LDLR* c.268G>A was earlier than that in the other patients, with a borderline effect in the statistical analysis (median, 44.2 years vs. 59.9 years, *p* = 0.060). Moreover, all of the five younger subjects with CAD (mean = 40.9 years) were male.

## 4. Discussion

FH is well known as the classic genetic cause of hypercholesterolemia, which leads to an increased risk of ASCVD. As such, substantial studies have focused on exploring the prevalence of FH genetic variants in several different CAD-associated high-risk populations. The present 10-year follow-up study specifically focused on a CAD-high-risk cohort of patients with high blood LDL-C levels (≥160 mg/dL) who underwent coronary angiography. A high FH prevalence rate (14.4%) was detected. FH genetic variation was shown to be associated with an increased risk of subsequent CV incidence or mortality. Furthermore, we demonstrated that FH genetic variants, especially *LDLR* c.986G>A and *LDLR* c.268G>A, had significant impacts on high blood LDL-C levels and early-onset CAD. Our results indicated that FH genetic variants could be an independent factor for predicting CV risks.

The incidence of FH genetic variants varied depending on inclusion criteria, ethnicities, or genotyping methodology. In this study, we found a 70-fold increase in the prevalence of FH pathogenic variants (14.4%, 41/285) through targeted sequencing of the patients with high blood LDL-C levels who underwent coronary angiography as compared with the general Chinese population (0.2%) [1,3]. When only patients with CAD were considered, the FH prevalence rate increased to 17.1% (36/211). Furthermore, the prevalence even doubled (31.4%, 11/35), with a >150-fold increase, in the patients with early-onset CAD. Our study showed that FH pathogenic variants carriers had significantly earlier CAD onset than non-carriers, despite the comparable blood LDL-C levels, which implied that FH pathogenic variation influenced the early development of CAD. We speculated that the long-term LDL exposure of FH variant carriers may result in a deleterious impact on the development of atherosclerosis. As they induce vascular inflammation, high blood LDL-C levels are well-known as a major risk factor for the initiation and promotion of atherosclerosis. Therefore, FH variant carriers with lifelong excess LDL-C accumulated in their arteries produce atheromas, leading to accelerated atherosclerosis and CVD development. Consistent with this, significantly higher carotid intima-media thickness (IMT) has been reported in child [18] and adult [19] patients with FH, indicating a higher cardiovascular risk. Recently, several significant potentially differently expressed genes and their functions, which were theoretically contributing to atherosclerosis development in FH patients, had been investigated to explore the mechanism of FH using various bioinformatic tools [20]. However, the specific molecular pathomechanisms of the atherosclerosis progression process in patients with FH are still not completely understood. Both the detailed effects and specific underlying mechanisms remain to be elucidated.

Previous studies reported that the total number of FH genetic variants identified in Caucasians [21,22,23,24] so far was much higher than that reported in the Han Chinese population [3,25]. In this study, the top three most frequent genetic variants in FH (LDLR c.1747C>T, LDLR c.986G>A, and APOB c.10579C>T) were consistent with previous studies in the Han Chinese population [3,25]. However, the prevalence of specific genes in our study might have been biased by the inclusion criteria, ethnicities, or genotyping methodology.

FH variation-caused high LDL-C level has been clinically related to high risks of ASCVD [4,5] and ischemic stroke [6,7]. Genetically, the presence of a pathogenic FH variant increases CVD risk more than threefold when compared with those with the same LDL-C level who do not carry such gene variants [26]. Our results also indicated that the FH variants were associated with an increased risk of CAD (OR = 3.17). Therefore, genetic testing provides additional prognostic and risk stratification values for CVD [8]. Furthermore, the severity of coronary and carotid atherosclerosis has been reported to be higher in those with monogenic FH than in those with high LDL-C levels due to a polygenic etiology [27]. A mean long-term follow-up of 6.1 years was conducted in our high-risk CAD cohort. Increased revascularization on follow-up was found in the subjects who were FH variant carriers. Moreover, our study showed that FH was the most important risk factor of increased incidence of CVD after controlling traditional CV risk factors. The risk was greater than threefold, which was the highest among all of the factors. Therefore, it is essential to identify FH in hypercholesteremic patients undergoing coronary angiography and provide stringent risk mitigation accordingly. In addition to FH, male sex, a smoking habit and diabetes, higher LDL-C levels also increased the mortality among patients with CAD symptoms. Our results were similar to those of the previous study [11], which demonstrated much higher prevalence rates of smoking and diabetes in the young subjects with CAD. As genetic factors are non-modifiable, our findings suggest that proactive control of blood pressure and glucose level and cessation of smoking might be a feasible method to curtail the CAD risks in patients with FH.

**Figure 1:**
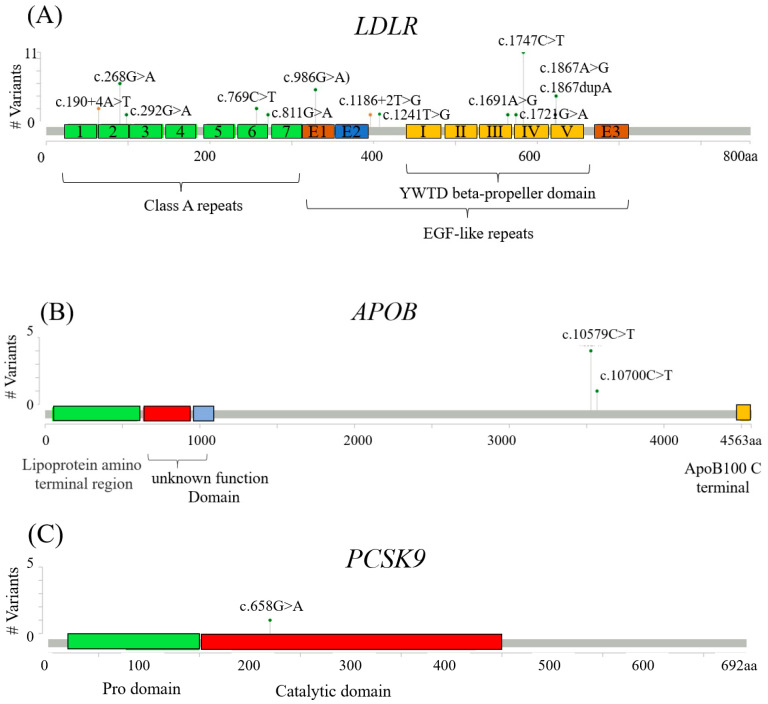
Lollipop plot showing the identified CAD subjects with FH variants relative to the schematic representation of the (**A**) *LDLR*, (**B**) *APOB*, and (**C**) *PCSK9* genes by the Mutation Mapper. The gray bar represents the amino acid positions (aa). The FH variant position is indicated with a lollipop circle, and the length of the line indicates the patient number of variants detected at that position. The vertical axis shows the frequency of the appearance of each variant. The colored boxes are specific functional domains.

Another important finding in this study was that the two FH genetic variants, *LDLR* c.986G>A and *LDLR* c.268G>A, were associated with high blood LDL-C levels and early CAD. The *LDLR* c.986 G>A variant is located in the epidermal growth factor (EGF) like repeats (Figure 1A), and the functional domain interacts with *PCSK9*, which promotes degradation of the *LDLR* [28]. Therefore, a variation in this site may affect the binding efficiency of *PCSK9*, which leads to loss or defect of functional hepatic receptors for the uptake and clearance of circulating LDL-C. The *LDLR* c.286G>A variant is located in class A repeats 2 (Figure 1A), which is required for maximal *APOB* binding to LDL [29]. Therefore, it may interfere with ligand binding and lead to severely high blood LDL-C levels [30]. Although genetic variation severity is a known concept within FH [18,26], our results indicated that FH genetic variants may exhibit differential effects on early-onset CAD and revascularization risks in patients with hypercholesterolemia undergoing coronary angiography. In particular, the *LDLR* c.986G>A and *LDLR* c.268G>A variants had the most significant impacts on high blood LDL-C levels and early-onset CAD. This FH genetic information might help identify high-risk patients with typical CAD symptoms for appropriate intervention. Furthermore, abundant evidence showed sex-related differences in CVD, demonstrating that men were at a higher risk of developing CVD than women [31,32]. In agreement with previous studies, our results showed that all five patients with early CAD were men among the *LDLR* c.986G>A/*LDLR* c.268G>A variant carriers.

The increased risk of FH prevalence in hypercholesterolemic patients with CAD symptoms strengthened the significant contribution of FH in patients undergoing coronary angiogram. We found that FH was identified in every 6–7 hypercholesterolemic CAD patients in this study cohort, a 20–30-fold increase compared with the general population. Increased prevalence of FH (4.5%) in early CAD patients [13] was accompanied by a surge of FH in the group with higher LDL-C. Additionally, in hypercholesterolemic patients with CAD symptoms, the FH carriers had a relatively higher risk of angiographically diagnosed CAD compared with patients of a similar age and with a similar LDL-C level. Therefore, it might be valuable to detect FH in hypercholesterolemic patients with CAD symptoms. Furthermore, more active intervention was recommended in hypercholesterolemic patients with CAD symptoms carrying FH variants due to their highest risk of myocardial infarction.

There were several limitations to this study. Firstly, we only detected the FH variations on three major genes (*LDLR*, *APOB*, and *PCSK9*) by targeted sequencing. Rare variants in other genes such as LDL receptor adaptor protein 1 (*LDLR*AP1) [1,8], ATP-binding cassette (ABC) hemitransporters, ABCG5 or ABCG8 [33] lysosomal acid lipase (LIPA) [34], and apolipoprotein E (APOE) [35] may also cause an FH-like phenotype. However, we were the first to identify two crucial variations on *LDLR* genes (*LDLR* c.986G>A and *LDLR* c.268G>A) in patients undergoing coronary angiography. Secondly, the relatively small numbers in our study cohort may not represent the general population. Nevertheless, we believed that FH genetic variations were crucial for early CAD events independent of traditional CV risk factors. Thirdly, the information on lipid-lowering medication prescriptions was lacking. Thus, we propose that future investigations should emphasize the ways that FH-associated genes might interact with the therapeutic effects of statins in this population.

In conclusion, we detected a high FH prevalence rate (14.4%) in our cohort of patients with high blood LDL-C levels undergoing coronary angiogram. Our results were the first to demonstrate that FH genetic variations, especially *LDLR* c.986G>A and *LDLR* c.268G>A variants, possess a significant impact on high blood LDL-C levels and early CAD. Thus, genetic variations could serve as an essential contributing factor for CV risk assessment in the future.

## Figures and Tables

**Table 1:** Comparison of the clinical characteristics of FH pathogenic variant carriers, *LDLR* c.986G>A/*LDLR* c.268G>A variant carriers ^†^ and FH pathogenic variant non-carriers in subjects with high blood LDL-C levels who underwent coronary angiography.

Variables	FH Pathogenic Variant Carriers	*LDLR* c.986G>A/c.268G>A Variant Carriers	FH Pathogenic Variant Non-Carriers	*p* Value ^1^	*p* Value ^2^
Number	41	9	244		
Age, years	59.2 ± 13.9	52.9 ± 13.2	59.7 ± 11.7	0.819	0.089
Men, n (%)	34(82.9%)	6(66.7%)	174(71.3%)	0.174	0.720
Body mass index, kg/m^2^	27.6 ± 4.3	27.4 ± 5.1	26.7 ± 3.8	0.178	0.593
Waist/Hip Ratio	0.94 ± 0.05	0.91 ± 0.07	0.94 ± 0.06	0.888	0.183
sBP, mmHg	131 ± 23	127 ± 18	132 ± 20	0.859	0.494
dBP, mmHg	77 ± 13	77 ± 14	77 ± 12	0.934	0.876
Triglycerides, mg/dL	180 ± 156	190 ± 80	178 ± 106	0.937	0.743
Cholesterol, mg/dL	239 ± 48	259 ± 34	239 ± 53	0.944	0.260
LDL-C, mg/dL	193 ± 27	213 ± 25	190 ± 30	0.633	0.026 *
HDL-C, mg/dL	44 ± 13	40 ± 13	46 ± 12	0.280	0.151
HbA1c, %	6.4 ± 1.5	6.2 ± 0.6	6.3 ± 1.3	0.727	0.849
Creatinine, mg/dL	1.2 ± 1.1	1.0 ± 0.2	1.4 ± 1.6	0.609	0.437
eGFR, ml/min/1.73 m^2^	76 ± 23	85 ± 22	75 ± 27	0.945	0.310
Smoking, n (%)	21(51.2%)	4(44.4%)	120(49.6%)	0.980	1.000
DM, n (%)	12(29.3%)	2(22.2%)	57(23.4%)	0.535	1.000
Hypertension, n (%)	27(65.9%)	6(66.7%)	146(60.1%)	0.598	1.000
CAD, n (%)	36(87.8%)	8(88.9%)	175(71.7%)	0.048 *	0.451
Early CAD, n (%)	11(26.8%)	5(55.6%)	24(9.8%)	0.005 **	0.001 **

*p* value ^1^: FH pathogenic variant carriers (n = 41) vs. FH pathogenic variant non-carriers (n = 244). *p* value ^2^: *LDLR* c.986G>A/*LDLR* c.268G>A variant carriers (n = 9) vs. FH pathogenic variant non-carriers (n = 244). ^†^: *LDLR* c.986G>A/c.268G>A variant carriers stand for those patients who carry either a *LDLR* c.986G>A heterozygotic variant or a *LDLR* c.268G>A heterozygotic variant. * *p* < 0.05, ** *p* < 0.01.

**Table 2:** Identified FH pathogenic gene loci in the study cohort.

Gene	Chromosome: Position	HGVSc	HGVSp	Variant Type	Clinical Significance	SNV ID	Number
*LDLR*	chr19:11227576	c.1747C>T	p.(His583Tyr))	missense	pathogenic	rs730882109	11
*LDLR*	chr19:11221373	c.986G>A	p.(Cys329Tyr)	missense	pathogenic	rs761954844	5
*LDLR*	chr19:11213417	c.268G>A	p.(Asp90Asn)	missense	pathogenic	rs749038326	4
*LDLR*	chr19:11230789	c.1867A>G	p.(Ile623Val)	missense	pathogenic	rs555292896	4
*LDLR*	chr19:11217315	c.769C>T	p.(Arg257Trp)	missense	pathogenic	rs200990725	2
*LDLR*	chr19:11224008	c.1241T>G	p.(Leu414Arg)	missense	likely pathogenic	rs748554592	1
*LDLR*	chr19:11213441	c.292G>A	p.(Gly98Ser)	missense	likely pathogenic	rs750474121	1
*LDLR*	chr19:11227550	c.1721G>A	p.(Arg574His)	missense	likely pathogenic	rs777188764	1
*LDLR*	chr19:11217357	c.811G>A	p.(Val271Ile)	missense	likely pathogenic	rs749220643	1
*LDLR*	chr19:11226874	c.1691A>G	p.(Asn564Ser)	missense	likely pathogenic	rs758194385	1
*LDLR*	chr19:11211025	c.190+4A>T		splice_region, intron	pathogenic	rs769446356	2
*LDLR*	chr19:11222317	c.1186+2T>G		splice donor	likely pathogenic	rs779921498	2
*LDLR*	chr19:11230789: 11230788	c.1867dupA	p.(Ile623AsnfsTer22)	frameshift	likely pathogenic	rs1555807206	1
*APOB*	chr2:21229161	c.10579C>T	p.(Arg3527Trp)	missense	pathogenic	rs144467873	4
*APOB*	chr2:21229040	c.10700C>T	p.(Thr3567Met)	missense	pathogenic	rs368278927	1
*PCSK9*	chr1:55518323	c.658G>A	p.(Ala220Thr)	missense splice_region	pathogenic	rs768795323	1

HGVSc: human genome variation society cDNA; HGVSp: human genome variation society protein.

**Table 3:** Revascularization on follow-up. Comparison of FH pathogenic variant carriers, *LDLR* c.986G>A/*LDLR* c.268G>A variant-carriers ^†^ and FH pathogenic variant non-carriers in subjects with high blood LDL-C levels who underwent coronary angiography.

Variables	FH Pathogenic Variant Carriers	LDLR c.986G>A/c.268G>A Variant Carriers *	FH Pathogenic Variant Non-Carriers	*p* Value ^1^	*p* Value ^2^
Number	41	9	244		
Revascularization on follow-up, n (%)	21(51.2%)	6(66.7%)	54(22.1%)	<0.001 **	0.007 **
PCI, n (%)	12(29.3%)	4(44.4%)	48(19.7%)	0.235	0.089
CABG, n (%)	8(19.5%)	2(22.2%)	5(2.0%)	<0.001 **	0.022 *

*p* value ^1^: FH pathogenic variants carriers (n = 41) vs. FH pathogenic variants non-carriers (n = 244). *p* value ^2^: *LDLR* c.986G>A/*LDLR* c.268G>A variant carriers (n = 9) vs. FH pathogenic variants non-carriers (n = 244). ^†^: *LDLR* c.986G>A/c.268G>A variant carriers stand for those patients who carry either a *LDLR* c.986G>A heterozygotic variant or a *LDLR* c.268G>A heterozygotic variant. * *p* < 0.05, ** *p* < 0.01.

**Table 4:** Association between the variables and the incidence of cardiovascular disease or mortality as determined by univariate and multivariate regression analyses in the study cohort (n = 285).

Variables	Univariate Analysis			Multivariate Analysis		
	Odds Ratio	(95% CI)	*p* Value	Odds Ratio	(95% CI)	*p* Value
Age, years	1.02	(1.00–1.04)	0.090			
Sex, men	2.40	(1.35–4.28)	0.003 **	2.23	(1.06–4.68)	0.034 *
Body mass index, kg/m^2^	1.04	(0.97–1.12)	0.253			
sBP, mmHg	1.01	(1.00–1.03)	0.097	1.02	(1.00–1.04)	0.081
dBP, mmHg	1.00	(0.98–1.02)	0.875	0.98	(0.95–1.01)	0.219
Triglycerides, mg/dL	1.00	(1.00–1.01)	0.123			
Cholesterol, mg/dL	1.00	(1.00–1.01)	0.262			
LDL-C, mg/dL	1.02	(1.00–1.03)	0.024 *	1.02	(1.00–1.03)	0.019 *
HDL-C, mg/dL	0.97	(0.95–1.00)	0.037 *			
HbA1c, %	1.67	(1.05–2.65)	0.032 *			
Creatinine, mg/dL	1.33	(0.91–1.94)	0.143			
eGFR, mL/min/1.73 m^2^	0.99	(0.98–1.00)	0.011 *	0.99	(0.98–1.00)	0.079
Smoking	2.51	(1.41–4.46)	0.002 **	2.09	(1.04–4.19)	0.039 *
DM	2.93	(1.32–6.49)	0.008 **	2.42	(1.02–5.73)	0.045 *
Hypertension	2.38	(1.36–4.16)	0.002 **			
FH genetic variation						
Non-carriers	Reference			Reference		
Carriers	3.29	(1.13–9.59)	0.029 *	3.17	(1.01–9.92)	0.047 *

Logistic regression. * *p* < 0.05, ** *p* < 0.01.; CI: confidence interval.

## Data Availability

Not applicable.

## Supplementary Materials

The following are available online at https://www.mdpi.com/article/10.3390/genes12091413/s1, Supplementary Table S1: Comparison clinical characteristics of FH pathogenic variant carriers, and FH pathogenic variant non-carriers in subjects with high blood LDL-C levels (≥190 mg/dL) who underwent coronary angiography. Supplementary Table S2: Comparison of the major adverse cardiac events (MACEs) between the FH variant carriers, LDLR c.986G>A/c.268G>A variant carriers†, and FH pathogenic variant non-carriers among the subjects with high blood LDL-C levels who underwent coronary angiography. Supplementary Table S3: Blood low-density lipoprotein cholesterol (LDL-C) levels and age of the men among carriers of the top five most frequent FH variants. Supplementary Figure S1: Age of coronary artery disease (CAD) onset in the subjects who were low-density lipoprotein cholesterol receptor (LDLR) c.986G>A/LDLR c.268G>A variant carriers (n = 8) and familial hypercholesterolemia (FH) variant non-carriers (n = 175). The centerline in the dot plot represents the median. The statistical analysis was performed with analysis of variance.
