# Supplementary material for: Familial Hypercholesterolemia Genetic Variations and Long-Term Cardiovascular Outcomes in Patients with Hypercholesterolemia Who Underwent Coronary Angiography
Source: Genes (Basel). 2021 Sep 14;12(9):1413. doi: 10.3390/genes12091413 (PMC8467756; doi:10.3390/genes12091413)
Supplement: Supplementary file 1 [file genes-12-01413-s001.zip › genes-1307257-supplementary.pdf]

Supplementary Table S1. Comparison clinical characteristics of FH pathogenic variant carriers, and FH pathogenic variant non-carriers in subjects with high blood LDL-C levels (≥ 190 mg/dL) who underwent coronary angiography.

Variables	FH pathogenic variant carriers	FH pathogenic variant non-carriers	*P* value
Number	18	89	
Age, years	56.11±12.88	59.04±11.27	0.328
Men, n (%)	15(83.3%)	61(68.5%)	0.329
Body mass index, kg/m^2	28.6±4.2	26.3±3.6	0.018*
Waist/Hip Ratio	0.94±0.06	0.93±0.05	0.634
sBP, mmHg	131±20	135±21	0.429
dBP, mmHg	81±13	79±13	0.615
Triglycerides, mg/dL	237±214	180±110	0.289
Cholesterol, mg/dL	250±61	262±64	0.433
LDL-C, mg/dL	215±25	218±36	0.794
HDL-C, mg/dL	44±14	47±12	0.414
HbA1c, %	6.25±1.62	6.31±1.43	0.888
Creatinine, mg/dL	1.07±0.16	1.42±1.78	0.420
eGFR, mL/min/1.73 m2	75±16	76±30	0.845
Smoking, n (%)	10(55.6%)	40(44.9%)	0.573
DM, n (%)	5(27.8%)	20(22.5%)	0.760
Hypertension, n (%)	10(55.6%)	50(56.2%)	1.000
CAD, n (%)	18(100.0%)	72(80.9%)	0.070
Early CAD, n (%)	8(44.4%)	13(14.6%)	0.008**

Chi-square test, n(%) or Independent t test, Mean±SD. *$p<0.05$, **$p<0.01$

Supplementary Table S2. Comparison of the major adverse cardiac events (MACEs) between the FH variant carriers, *LDLR* c.986G>A/c.268G>A variant carriers†, and FH pathogenic variant non-carriers among the subjects with high blood LDL-C levels who underwent coronary angiography.

Variable	FH pathogenic variant carriers	*LDLR* c.986G>A/c.268G>A variant carriers	FH pathogenic variant non-carriers	*p* Value[1]	*p* value[2]
Number	41	9	244		
**All-cause death, n (%)**	7(17.1%)	1(11.1%)	34(13.9%)	0.772	1.000
CV death, n (%)	1(2.4%)	1(11.1%)	7(2.9%)	1.000	0.255
Non-CV death, n (%)	6(14.6%)	0(0.0%)	27(11.1%)	0.596	0.603
**Nonfatal MI, n (%)**	3(7.3%)	1(11.1%)	10(4.1%)	0.334	0.410
Q-Wave, n (%)	2(4.9%)	1(11.1%)	4(1.6%)	0.167	0.208
Non-Q-Wave, n (%)	1(2.4%)	0(0.0%)	6(2.5%)	1.000	1.000
**Nonfatal stroke, n (%)**	0(0.0%)	0(0.0%)	1(0.4%)	1.000	1.000
Ischemic, n (%)	0(0.0%)	0(0.0%)	1(0.4%)	1.000	1.000
Hemorrhagic, n (%)	0(0.0%)	0(0.0%)	0(0.0%)	--	--

p Value[1]: FH pathogenic variant carriers (n=41) compared with FH pathogenic variant non-carriers (n=244). *p* Value[2]: *LDLR* c.986G>A/*LDLR* c.268G>A variant carriers (n=9) compared with FH pathogenic variant non-carriers (n=244). †: *LDLR* c.986G>A/ c.268G>A variant carriers stand for those patients who carry either a *LDLR* c.986G>A heterozygotic variant or a *LDLR* c.268G>A heterozygotic variant.

Supplementary Table S3. Blood low-density lipoprotein cholesterol (LDL-C) levels and age of the men among carriers of the top five most frequent FH variants.

**Variant**	**LDL-C, mg/dL**	**Age, years**
***LDLR* c.986G>A**	**214±25**	55.7±11.9
***LDLR* c.268G>A**	**212±29**	39.0±1.7
LDLR c.1747C>T	189±22	62.3±13.8
LDLR c.1867A>G	179±10	69.7±22.2
APOB c.10579C>T	182±10	59.3±16.5

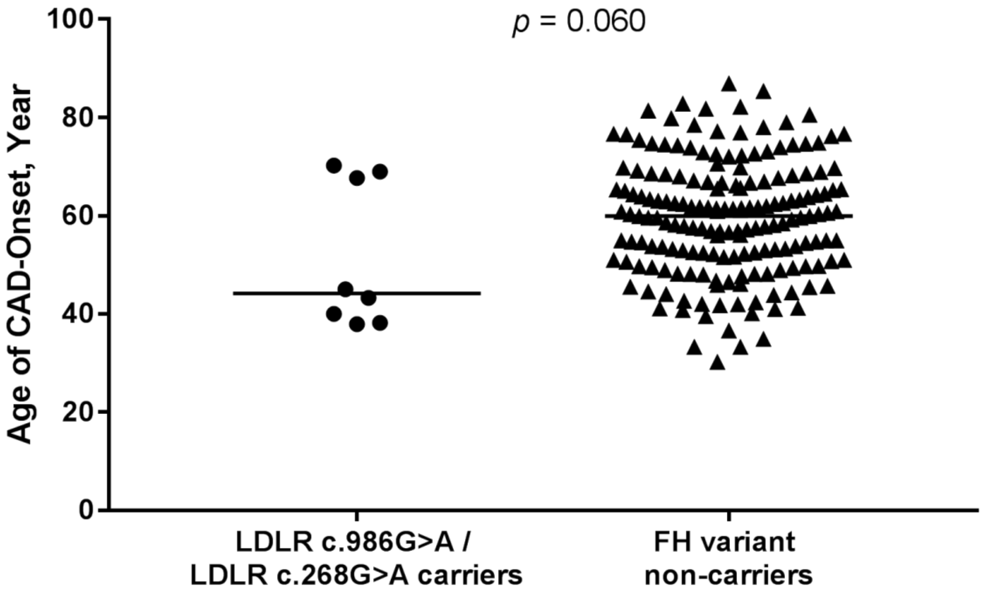

Supplementary Figure S1. Age of coronary artery disease (CAD) onset in the subjects who were low-density lipoprotein cholesterol receptor (*LDLR*) c.986G>A/*LDLR* c.268G>A variant carriers (n=8) and familial hypercholesterolemia (FH) variant non-carriers (n=175). The centerline in the dot plot represents the median. The statistical analysis was performed with analysis of variance.
